# Protein Kinase C subtype δ interacts with Venezuelan equine encephalitis virus capsid protein and regulates viral RNA binding through modulation of capsid phosphorylation

**DOI:** 10.1371/journal.ppat.1008282

**Published:** 2020-03-09

**Authors:** Brian D. Carey, Ivan Akhrymuk, Bibha Dahal, Chelsea L. Pinkham, Nicole Bracci, Sarah Finstuen-Magro, Shih-Chao Lin, Caitlin W. Lehman, Kevin J. Sokoloski, Kylene Kehn-Hall

**Affiliations:** 1 National Center for Biodefense and Infectious Diseases, School of Systems Biology, George Mason University, Manassas, Virginia, United States of America; 2 Department of Microbiology and Immunology, and the Center for Predictive Medicine for Biodefense and Emerging Infectious Diseases, University of Louisville School of Medicine, Louisville, Kentucky, United States of America; University of North Carolina at Chapel Hill, UNITED STATES

## Abstract

Protein phosphorylation plays an important role during the life cycle of many viruses. Venezuelan equine encephalitis virus (VEEV) capsid protein has recently been shown to be phosphorylated at four residues. Here those studies are extended to determine the kinase responsible for phosphorylation and the importance of capsid phosphorylation during the viral life cycle. Phosphorylation site prediction software suggests that Protein Kinase C (PKC) is responsible for phosphorylation of VEEV capsid. VEEV capsid co-immunoprecipitated with PKCδ, but not other PKC isoforms and siRNA knockdown of PKCδ caused a decrease in viral replication. Furthermore, knockdown of PKCδ by siRNA decreased capsid phosphorylation. A virus with capsid phosphorylation sites mutated to alanine (VEEV CPD) displayed a lower genomic copy to pfu ratio than the parental virus; suggesting more efficient viral assembly and more infectious particles being released. RNA:capsid binding was significantly increased in the mutant virus, confirming these results. Finally, VEEV CPD is attenuated in a mouse model of infection, with mice showing increased survival and decreased clinical signs as compared to mice infected with the parental virus. Collectively our data support a model in which PKCδ mediated capsid phosphorylation regulates viral RNA binding and assembly, significantly impacting viral pathogenesis.

## Author summary

Venezuelan equine encephalitis virus causes flu-like symptoms that can lead to severe encephalitic disease and sometimes death. Understanding the molecular mechanisms of host-virus interactions can aid in the design of intervention strategies in a field that is severely lacking FDA-approved therapeutics and vaccines. The significance of our research is identifying the mechanism behind VEEV capsid phosphorylation and unveiling its importance during infection and disease development. We have discovered that PKCδ modulates VEEV capsid phosphorylation and that phosphorylation of VEEV capsid is important for viral RNA binding, assembly, and pathogenesis. Thus, capsid phosphorylation events could represent a potential target in the development of vaccine design strategies.

## Introduction

Epidemics or epizootics caused by mosquito-borne Venezuelan equine encephalitis virus (VEEV) have resulted in devastating outbreaks of VEE involving equine and human cases in Columbia, Venezuela, and Trinidad since the 1930s [1]. VEEV is a member of the *Togaviridae* family of viruses and is in the new-world clade of the genus *alphavirus*. The virus causes flu-like symptoms including myalgia, fever, fatigue, nausea, and pharyngitis in humans. In up to 14%, of cases, severe neurological complications can occur due to encephalitis, including seizures, confusion, blurred vision and coma. Progression to encephalitis can lead to long lasting neurological deficits and about 1% of cases are fatal [2–5].

The alphavirus virion is enveloped with viral glycoproteins, E1 and E2, incorporated into the membrane. The genome is approximately 11.4kb and is positive sense single stranded RNA encoding two open reading frames. Four non-structural proteins (nsP1-4) are encoded by the first reading frame which begins at the 5’ end of the genome. The second open reading frame is controlled by a 26S promoter on the negative strand and encodes for the structural proteins including capsid, E2, 6K, and E1. A small E3 protein is formed during post-translational modification of the precursor E2 protein [6].

VEEV capsid is made up of 275 amino acid residues and is composed of two independent domains: N-terminal and C-terminal [7]. The main functions of capsid are to bind viral RNA, assist in replication and RNA packaging of the virus, and to subvert antiviral activities [8]. Binding of viral RNA is achieved through a highly conserved region between amino acid residues 76 and 116 [9]. Capsid also induces cytopathogenicity by shutting off host transcription. This function has been mapped to the N-terminal domain and is independent of the RNA binding domain [10]. Nuclear export and nuclear localization signals (NES and NLS, respectively) have been found in the N-terminal domain. Furthermore, capsid has been shown to form a complex with host nuclear export karyopherin CRM1 and the nuclear import karyopherins importin α/β1 [11]. It has additionally been shown that this complex blocks the nuclear pore and prevents nuclear trafficking [11]. The C-terminus is highly conserved amongst alphaviruses and functions as a protease [12]. The protease domain is responsible for cleaving itself from the translating polyprotein [13].

Protein phosphorylation is an essential post-translational modification and is one of the most abundant post-translational modifications in eukaryotes [14]. It is regulated by two classes of enzymes: kinases, which phosphorylate substrates; and phosphatases, which dephosphorylate substrates [15,16]. Protein phosphorylation assists in the life cycle of many viruses, such as the phosphorylation and subsequent cytoplasmic translocation of the severe acute respiratory syndrome coronavirus (SARS-CoV) N protein, mediated by the kinases ERK1/2 and GSK3 [17,18]. Additionally, some viruses encode kinases, such as the herpes simplex virus-1 (HSV-1) Us3 kinase, which is crucial for viral pathogenicity by regulating HSV-1 dUTPase activity [19]. Moreover, the phosphorylation of alphavirus proteins have also been shown to be key for functionality, demonstrated by phosphorylation of Sindbis virus (SINV) E2 glycoprotein which is necessary for viral maturation [20] and the hypervariable domain of nsP3 which is important for minus strand synthesis [21–23]. We have recently shown that VEEV capsid protein is phosphorylated on T93, T108, S124, and T127 residues and that phosphorylation is in part regulated by the phosphatase Protein Phosphatase 1α (PP1α) [24]. Here, we extended our study to determine the kinase responsible for capsid phosphorylation and to further elucidate the importance of phosphorylation for viral replication and pathogenesis.

## Results

### VEEV capsid co-immunoprecipitates with PKCδ, but not other PKC isoforms

Phospho-prediction site server, NetPhos 3.1 is an online tool that predicts phosphorylation sites in eukaryotic proteins using ensembles of neural networks. Analysis of the primary amino acid sequence of capsid by NetPhos 3.1 [25] resulted in a list of potential phosphorylation sites on capsid based on known kinase consensus sequences (Table 1). Any potential sites of tyrosine phosphorylation were removed as previous results indicated capsid was phosphorylated on serine and threonine residues. Next, any residues with a score below 0.500 were removed from the analysis because scores below 0.500 indicate less than 50% confidence that the site is actually phosphorylated. The software also listed potential kinases for each residue. Our previous data showed that capsid is phosphorylated on T93, T108, S124, and T127 [24]. NetPhos 3.1 predicted an unknown kinase and protein kinase C (PKC) to be the kinases responsible for phosphorylation of T93, T108, and S124. T127 was not predicted to be phosphorylated by the NetPhos 3.1 software with any statistical significance.

**Table 1:** Predicted Sites of Phosphorylation on VEEV capsid^3^.

**Sequence**	**Amino Acid**	**Position #**	**Score**^1^	**Potential Kinase**
ELTRSMANL	Serine	44	0.561	PKA
ELTRSMANL	Serine	44	0.506	DNAPK
MANLTFKQR	Threonine	49	0.887	PKC
PEGPSAKKP	Serine	62	0.972	Unknown
PEGPSAKKP	Serine	62	0.705	PKC
KKEASQKQK	Serine	71	0.981	Unknown
KKEASQKQK	Serine	71	0.781	PKC
KKEASQKQK	Serine	71	0.582	DNAPK
**KKAKTGPPN**^2^	**Threonine**	**93**	**0.782**	**PKC**
**NKKKTNKKP**	**Threonine**	**108**	**0.905**	**PKC**
**MKLESDKTF**	**Serine**	**124**	**0.738**	**Unknown**
**MKLESDKTF**	**Serine**	**124**	**0.683**	**PKC**
AALKTKKAS	Threonine	167	0.931	PKC
TKKASKYDL	Serine	171	0.998	Unknown
TKKASKYDL	Serine	171	0.725	PKA
MRADTFKYT	Threonine	188	0.943	Unknown
MRADTFKYT	Threonine	188	0.829	PKC
TFKYTHEKP	Threonine	192	0.537	PKC
QGYYSWHHG	Serine	201	0.762	Unknown
NGRFTVPKG	Threonine	215	0.658	PKC
AKGDSGRPI	Serine	226	0.926	Unknown
EKGVTVKYT	Threonine	264	0.88	PKC

^1^Score is the confidence that the software has of the site being a true phosphorylation site. Values above 0.500 are considered above the threshold

^2^Bolded information indicates sites that were previously experimentally shown to be phosphorylated [24].

^3^Predicted tyrosine phosphorylations are not included.

**Fig 1:**
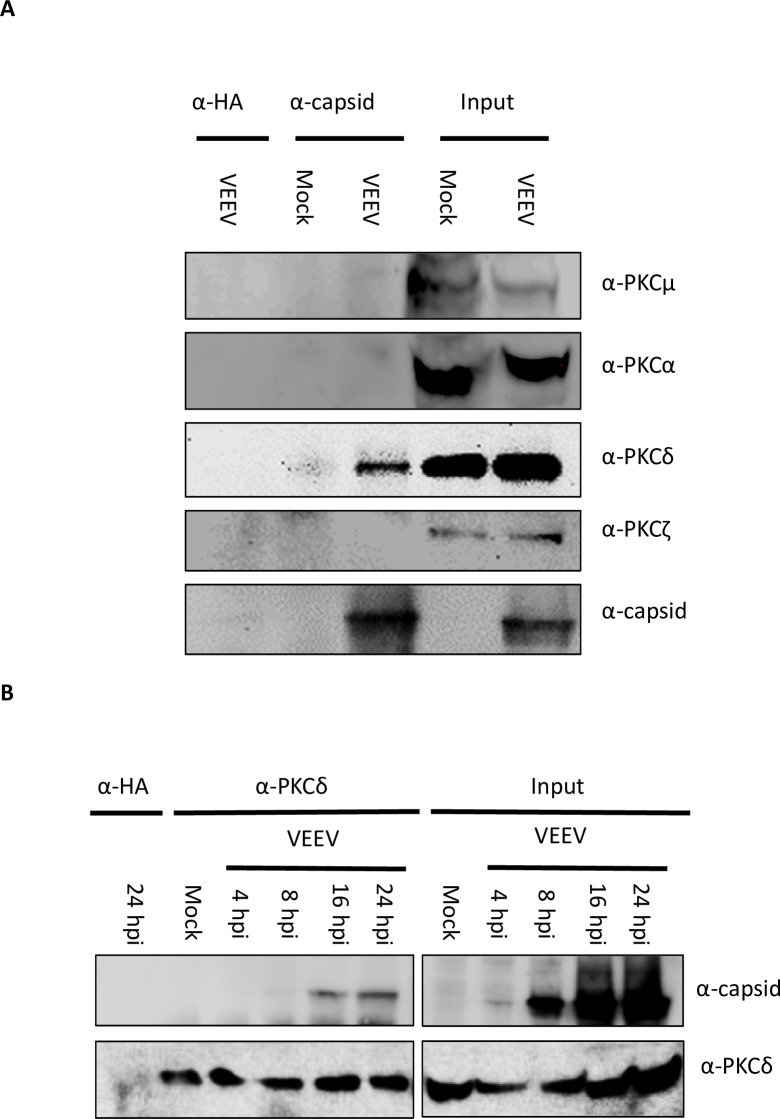
VEEV capsid co-immunoprecipitates with PKCδ, but not other PKC family members. A) Vero cells were mock-infected or infected with VEEV TC-83 (MOI of 1.0) for 24 hours. Cells were lysed and 1 μg of either α-HA or α-capsid antibody was added to 1 mg of protein lysate. Protein complexes were bound to Protein G Dynabeads, and samples were run on SDS-PAGE and western blot analysis was performed for VEEV capsid and each PKC isoform shown. Images are representative of 3 biological replicates. B) Vero cells were mock-infected or infected with VEEV TC-83 (MOI of 1.0) and collected at the indicated time points. Cells were lysed and 1 μg of either α-HA or α-PKCδ antibody was added to 1 mg of protein lysate. Protein complexes were bound to Protein G Dynabeads, samples were run on SDS-PAGE and western blot analysis was performed for capsid and PKCδ. Images are representative of 3 biological replicates.

**Fig 2:**
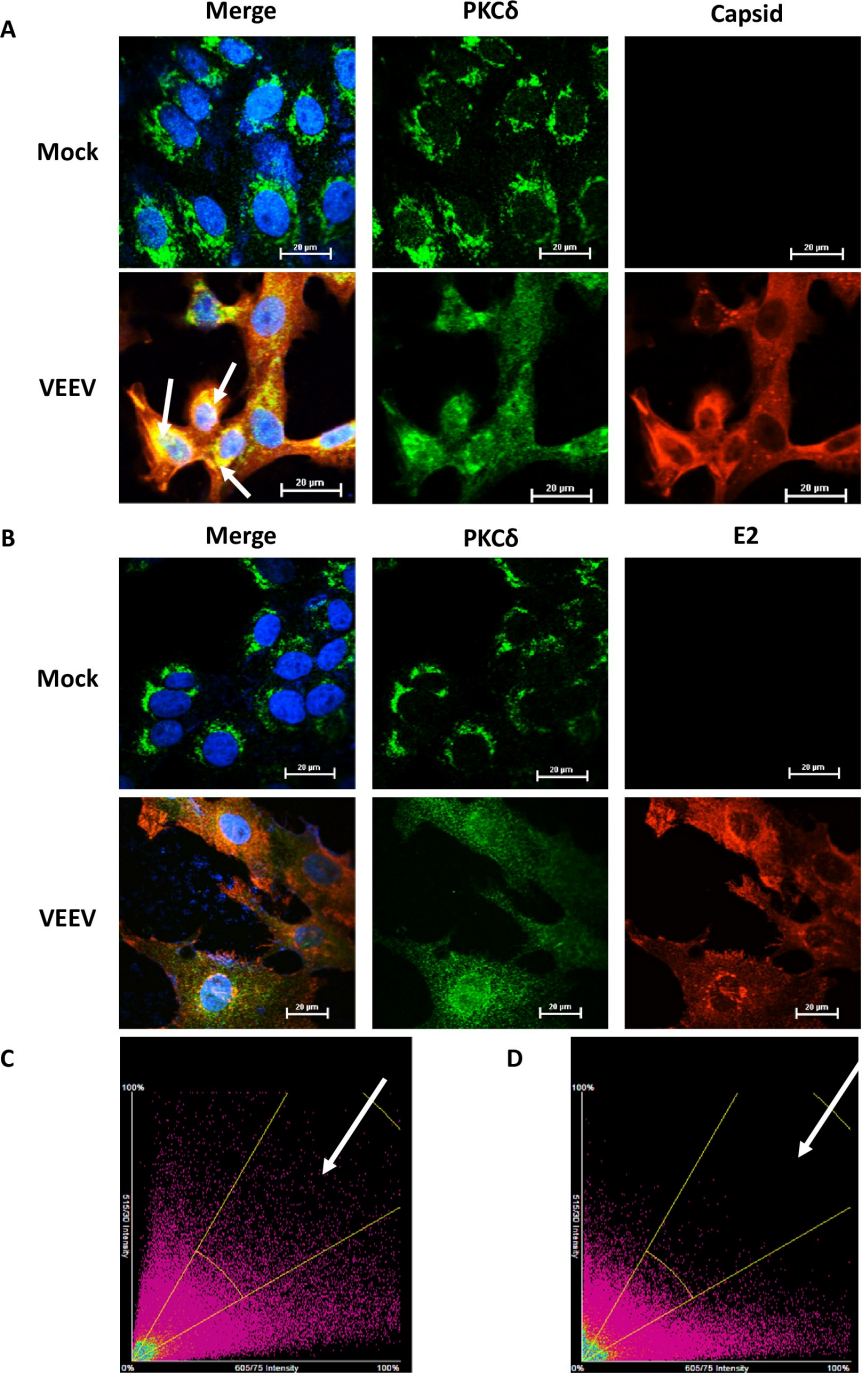
PKCδ co-localizes with VEEV capsid but not VEEV E2. A) Representative confocal microscopy images of mock-infected cells or cells infected with VEEV TC-83 (MOI 1.0) for 16 hours. Blue indicates the nucleus (DAPI), green indicates PKCδ, and red indicates VEEV capsid. B) Representative confocal microscopy images of either mock-infected cells or cells infected with VEEV TC-83 (MOI 1.0) for 16 hours. Blue indicates the nucleus (DAPI), green indicates PKCδ, and red indicates VEEV E2. C) Scatter plot of z-stack analysis from confocal microscopy for the entire image. X-axis = capsid, y-axis = PKCδ. Yellow gate shows increased pixel intensity vs. E2. Pearson’s correlation was calculated on regions of interest for capsid/PKCδ co-localization (arrowheads in A). Pearson’s correlation = 0.8603, n = 208 total slices. D) Scatter plot of z-stack analysis from confocal microscopy for the entire image. X-axis = capsid, y-axis = PKCδ. Yellow gate shows decreased pixel intensity vs. capsid. Pearson’s correlation was calculated on regions of interest for capsid/PKCδ co-localization. Pearson’s correlation = 0.3138, n = 118 total slices. p<0.0001 for both C & D.

In order to determine if PKC associates with capsid, co-immunoprecipitation experiments were performed. Vero cells were infected with VEEV TC-83 (a live attenuated vaccine strain of VEEV) or mock infected. Following infection, cells were lysed and samples were immunoprecipitated with an antibody against VEEV capsid. Western blot analysis was performed on immunoprecipitated samples with antibodies against PKC isoforms α, δ, μ, and ζ. Analysis demonstrated an association between PKCδ and capsid but no other PKC isoforms tested (Fig 1A). PKCδ was also found to interact with STAT3, a known PKCδ interacting partner [26] (S1A Fig). A reverse co-immunoprecipitaion confirmed the interaction of PKCδ with capsid (Fig 1B), but PKCδ did not interact with the viral 6K/TF protein (S1A Fig). To determine at what point of the viral life cycle this interaction was most prevalent, cells were infected with VEEV TC-83 and collected at 4, 8, 16, and 24 hours post infection (hpi). Samples were immunoprecipitated with an antibody against PKCδ and western blot analysis for VEEV capsid was performed. Results indicated the association between capsid and PKCδ is highest at 16 and 24 hpi (Fig 1B) with a slight interaction observed at 8 hpi. The detection at later time points could, however, be due to increased levels of capsid at later time points making the interaction easier to detect. Furthermore, confocal microscopy was performed to visualize co-localization between VEEV capsid and PKCδ. Cells were either mock infected or infected with VEEV TC-83 and incubated for 16 hours. Cells were fixed, permeabilized, and stained with antibodies against PKCδ and either VEEV capsid (Fig 2A) or VEEV E2 (Fig 2B). Consistent with immunoprecipitation results, a clear co-localization between VEEV capsid and PKCδ was detected. Co-localization analysis of z-stack images was performed and a scatter plot was created comparing the intensities of the red pixels (x-axis) with the green pixels (y-axis) (Fig 2C). The scatter plot shows the intensities of every pixel within the image, the region within the yellow gate and indicated by the arrow corresponds to the regions of interest of which the Pearson’s correlation was calculated (arrows in Fig 2A). A Pearson’s correlation at the regions of interest was 0.86 indicating a relatively strong correlation. Co-localization analysis of E2 and PKCδ (Fig 2D) using the same gating as the capsid:PKCδ analysis did not show a strong correlation of co-localization with a Pearson’s correlation of 0.31. Interestingly, localization of PKCδ in uninfected cells was fairly compact and perinuclear, suggesting localization in the ER/Golgi. However, after VEEV infection PKCδ localization displayed a more diffuse phenotype. Collectively, these results suggest that VEEV capsid interacts with PKCδ.

### PKCδ modulates the phosphorylation of VEEV capsid

**Fig 3:**
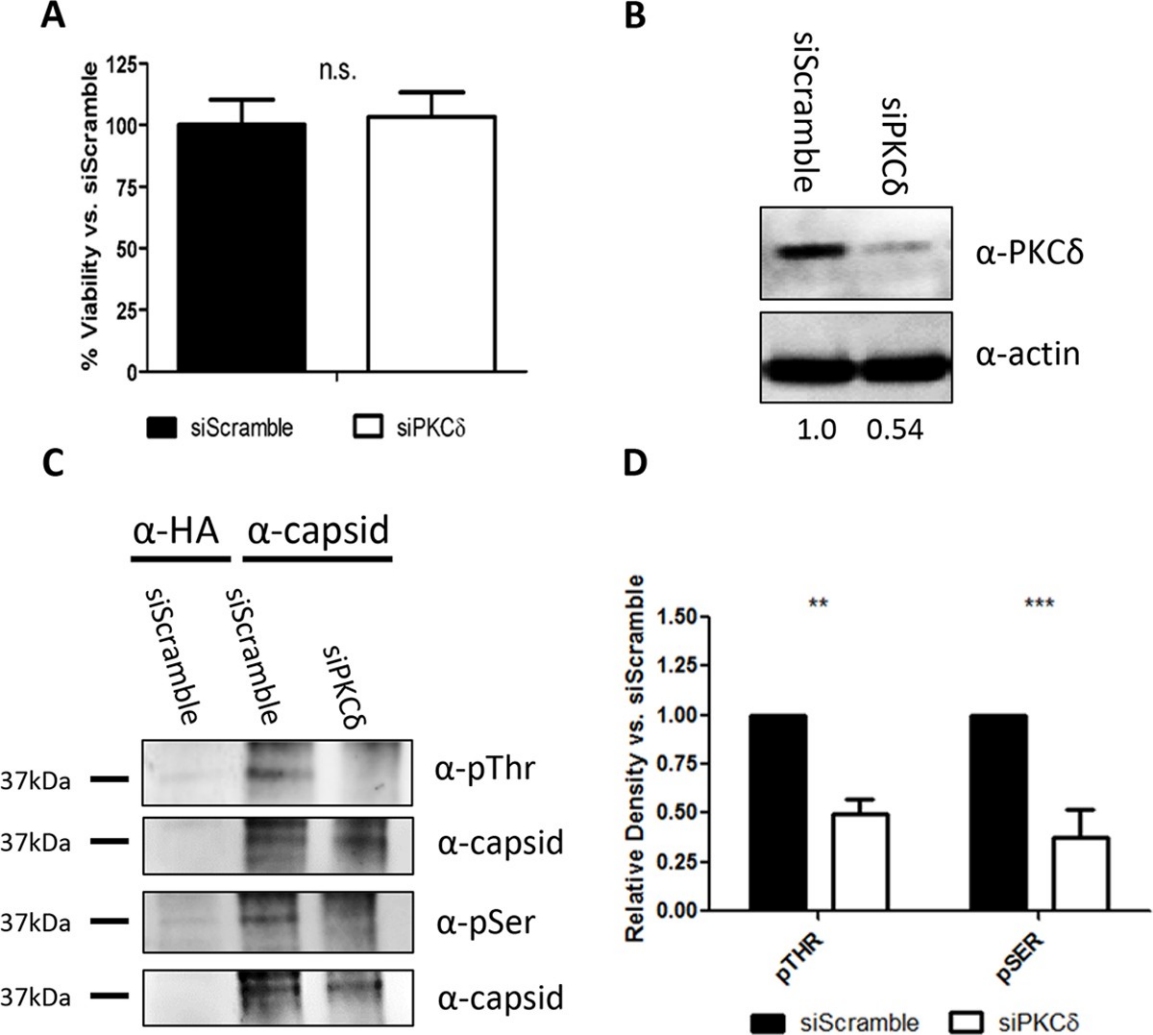
siRNA Knockdown of PKCδ decreases phosphorylation of VEEV capsid. A) U87MG cells were transfected with 50 nM of the indicated siRNA and incubated for 72 hours. Cell viability was measured using Cell Titer-Glo assay from Promega. Luminescence was measured and normalized to siRNA scramble data. Values are average of 8 biological replicates. B) Western blots probing for PKCδ and actin from cell lysates treated with the indicated siRNA. Band density was analyzed on BioRad Quantity One software and normalized to actin. Normalized values were calculated relative to siScramble and values are below the actin blot in their respective lane. Images are representative of 3 biological replicates. C) U87MG cells were transfected with siRNA against PKCδ or a scrambled control and incubated for 72 hours. Cells were transfected with a plasmid expressing the VEEV structural polyprotein and incubated for 48 hours. Cells were collected, lysed, and immunoprecipitated with α-HA or α-VEEV capsid antibodies. Protein complexes were bound to Protein G Dynabeads, samples were run on SDS-PAGE, and western blot analysis was performed for phospho-Ser or phospho-Thr residues. D) Western blot band density was analyzed on BioRad Quantity One software and normalized to capsid. Normalized values were calculated relative to siScramble transfected cells. Quantitation was performed for 3 biological replicates.

PKCδ is a serine/threonine kinase and our previous work indicated VEEV capsid is phosphorylated on specific serine/threonine residues [24]. We set out to determine if PKCδ is responsible for the phosphorylation of VEEV capsid. Cells were treated with siRNA targeting either PKCδ or a scrambled control, and then transfected with a plasmid expressing the structural polyprotein of VEEV. Following 48 hours of incubation, cells were collected, lysed, and samples were immunoprecipitated with an antibody against VEEV capsid. Anti-phospho-serine and anti-phospho-threonine antibodies were used to detect phosphorylation via western blot analysis. PKCδ siRNA had no effect on cell viability (Fig 3A) and PKCδ protein expression was reduced by ~50% (Fig 3B). Capsid threonine phosphorylation was decreased by 50% and serine phosphorylation by 60% following PKCδ siRNA treatment (Fig 3C & 3D). Loss of PKCδ also resulted in decreased phosphorylation of STAT3 serine 727, which is a known PKCδ substrate [26] (S1B Fig), further confirming the specificity of our siRNA results. These data suggest that PKCδ is involved in the phosphorylation of VEEV capsid.

### Loss of PKCδ decreases VEEV replication

**Fig 4:**
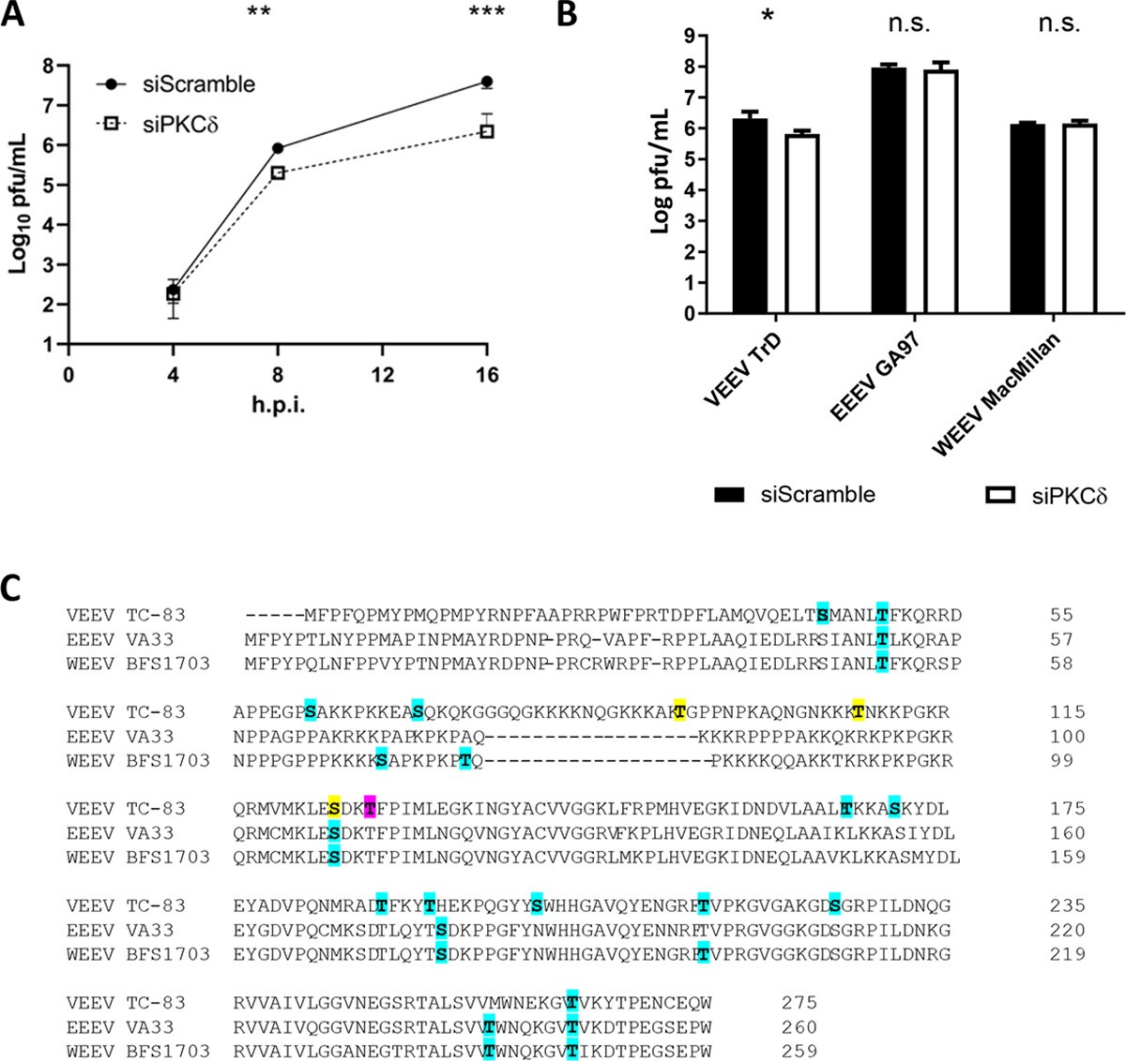
Inhibition of PKCδ causes a decrease in viral replication. U87MG cells were transfected with 50 nM scramble control or PKCδ siRNAs. Seventy-two hours post-transfection, cells were infected with A) VEEV TC-83 (MOI 0.1) or B) VEEV TrD, EEEV GA97, or WEEV 1930 California (MOI 0.1) and viral supernatants collected at 4, 8, and 16 hpi (panel A) or 16 hpi (panel B) for viral titer determination via plaque assay. C) An alignment of the primary amino acid sequence of VEEV, EEEV, and WEEV is displayed. All residues predicted (but not experimentally verified) are bolded and highlighted in cyan. Residues that were predicted and experimentally verified to be phosphorylated are bolded and highlighted in yellow. Thr 127 which was experimentally shown, but not predicted, is bolded and highlighted in pink. Data is the average of 3 biological replicates ± standard deviation * = p<0.05, ** = p<0.005, *** = p<0.0005.

In order to determine if the decrease in phosphorylation is important for VEEV replication, viral production was measured after siRNA knockdown of PKCδ. Transfection of siRNA against PKCδ caused a significant time-dependent decrease in VEEV TC-83 viral titers (Fig 4A) with more than a one log decrease at 16 hpi. Similar results were observed with virulent BSL-3 VEEV TrD, with ~ 1 log reduction of viral replication at 16 hpi. However, loss of PKC had no impact on eastern equine encephalitis virus (EEEV) or western equine encephalitis virus (WEEV) (Fig 4B). An alignment of the primary amino acid sequences for the capsid proteins of VEEV, EEEV, and WEEV showed that while both serine 124 and threonine 127 are conserved amongst all three viruses only serine 124 is predicted to be phosphorylated in all three viruses. Interestingly, threonine 127 was also not predicted to be phosphorylated in VEEV capsid, but was detected by LC-MS/MS analysis (24). Threonine 93 and 108 were not conserved in EEEV or WEEV. These data suggest that PKCδ is important for VEEV replication, but not related alphaviruses.

### Capsid phosphorylation-deficient (CPD) mutant virus has reduced capsid phosphorylation and is insensitive to the loss of PKCδ

**Fig 5:**
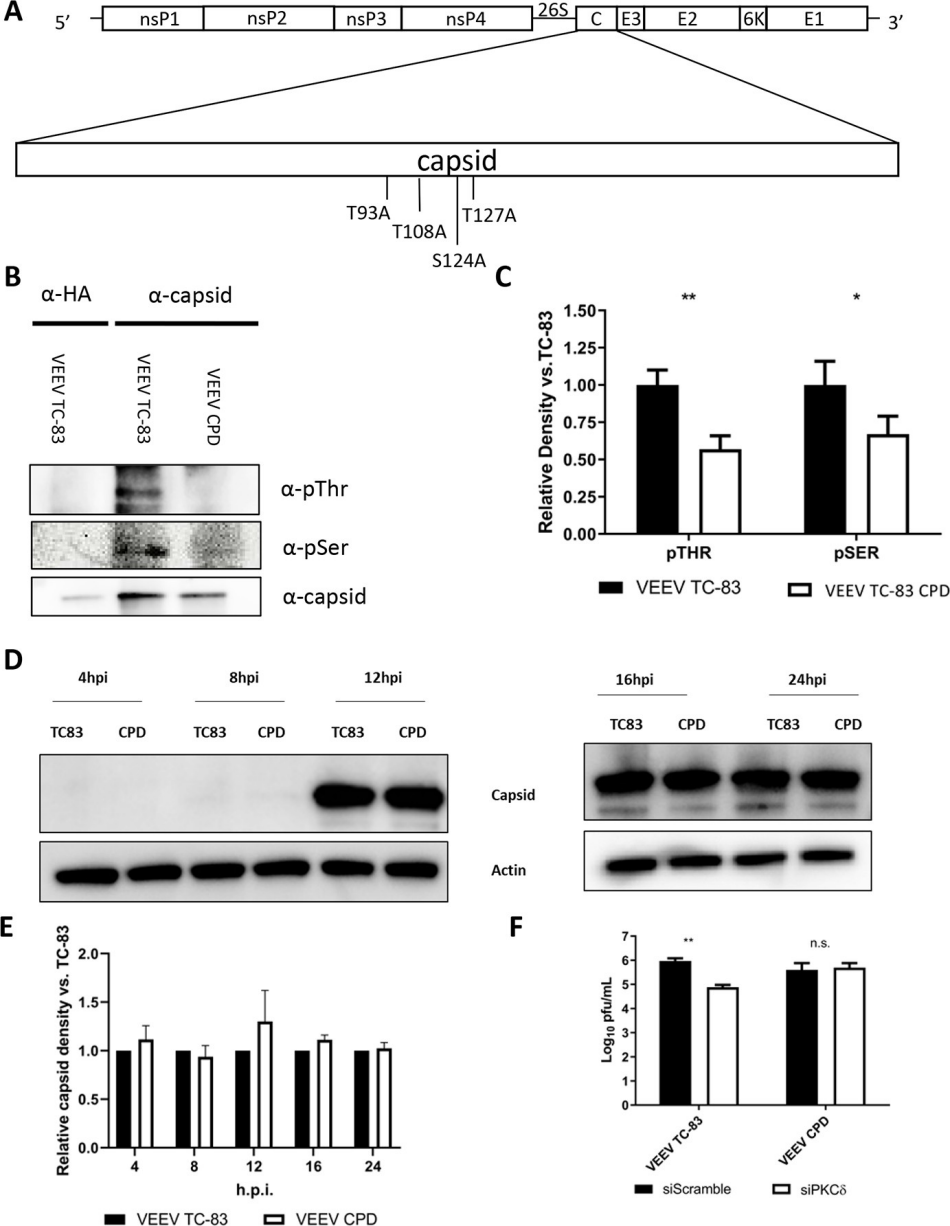
VEEV CPD shows decreased phosphorylation of capsid and replication is not sensitive to loss of PKCδ. A) Schematic of the VEEV genome indicating the alanine substitutions made at T93, T108, S124, and T127. B) Vero cells were infected with either VEEV TC-83 or VEEV CPD (MOI 1.0). Cells were collected at 16 hpi, lysed, and immunoprecipitated with α-HA or α-VEEV capsid antibodies. Protein complexes were bound to Protein G Dynabeads, samples were run on SDS-PAGE, and western blot analysis was performed for phospho-Ser or phospho-Thr residues. Images are representative of 3 biological replicates. C) Western blot band density was analyzed on BioRad Quantity One software and normalized to capsid. Normalized values were calculated relative to TC-83 infected cells. Quantitation was performed for 3 biological replicates. * = p<0.05 D) Vero cells were infected with either VEEV TC-83 or VEEV CPD (MOI 1.0). Cells were collected at the indicated time points, lysed, samples were run on SDS-PAGE, and western blot analysis was performed for capsid and actin. E) Band density from panel D was analyzed on BioRad Quantity One software and normalized to actin. Normalized values were calculated relative to TC-83 infected cells respective to each time point (for example, 16hpi CPD is relative to 16hpi TC-83 and 24hpi CPD is relative to 24hpi TC-83). Images were split into early and late time points to allow for longer exposure on 4 and 8 hpi without over exposing the later time points. F) U87MG cells were transfected with 50 nM scramble control or PKCδ siRNAs. Seventy-two hours post-transfection, cells were infected with either VEEV TC-83 or VEEV CPD (MOI 0.1) and viral supernatants collected at 16 hpi for viral titer determination via plaque assay. Values are an average of 3 biological replicates ± standard deviation * = p<0.05, ** = p<0.005.

To further investigate the importance of the phosphorylation of VEEV capsid, we designed a capsid phosphorylation-deficient (CPD) mutant virus using the VEEV TC-83 backbone. We have previously shown that capsid is phosphorylated on four residues in the RNA-binding domain [24]. VEEV CPD was produced by mutating the previously identified phosphorylation sites to alanine (T93A, T108A, S124A, and T127A) (Fig 5A). Cells infected with VEEV CPD showed decreased capsid phosphorylation on both threonine and serine residues (Fig 5B & 5C) with no significant difference in capsid expression (Fig 5D & 5E).

To examine the link between VEEV CPD and PKCδ, cells were treated with siRNA targeting PKCδ or a scrambled control and then infected with either VEEV TC-83 or VEEV CPD. VEEV CPD replication was not affected by PKCδ knockdown while VEEV TC-83 exhibited a similar decrease in titer as with earlier siRNA experiments (Fig 5F). These results indicate that replication of VEEV CPD is not sensitive to the loss of PKCδ suggesting that PKCδ plays a role in the phosphorylation of capsid.

### VEEV CPD is more infectious than VEEV TC-83

**Fig 6:**
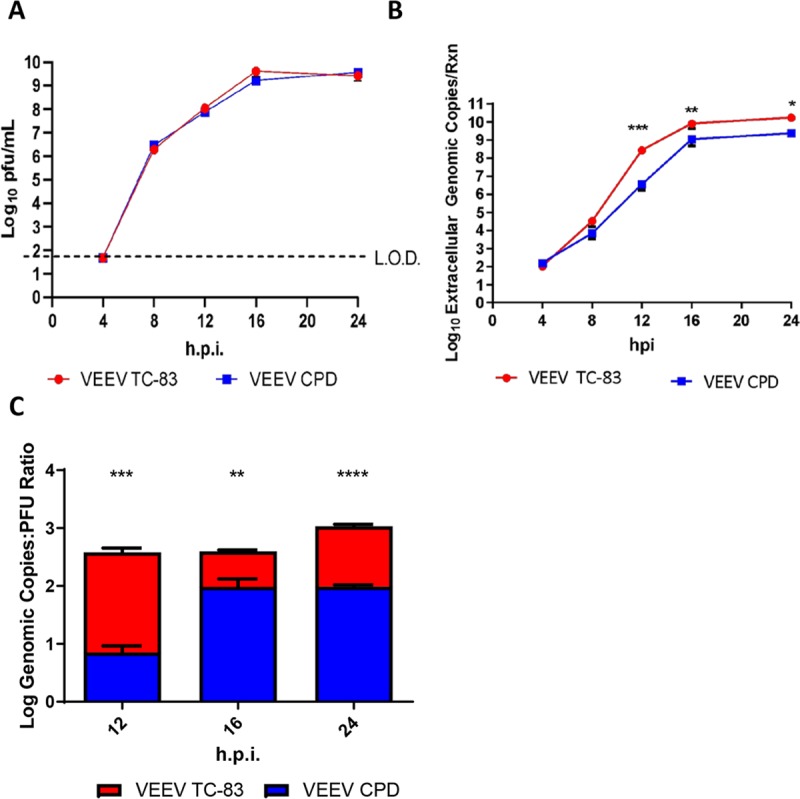
VEEV CPD is more infectious than VEEV TC-83. Vero cells were infected with either VEEV TC-83 or VEEV CPD (MOI 0.1) and viral supernatants collected at the indicated time points for viral titer determination via A) plaque assay or B) RT-qPCR. C). Genomic copies:PFU ratio was calculated at the indicated time points by converting RT-qPCR genomic copy per reaction data to genomic copies per milliliter. Genomic copies per milliliter were then divided by PFU/mL of the respective time point to obtain the genomic copies to PFU ratio. Values are an average of 3 biological replicates ± standard deviation. * = p<0.05, ** = p<0.005, *** = p<0.0005, **** = p<0.0001.

To determine the impact of capsid phosphorylation on viral replication, viral growth kinetics of VEEV TC83 and VEEV CPD were measured at 4, 8, 12, 16, and 24 hpi in Vero cells. Growth kinetics were also observed in human primary astrocytes, given that astrocytes are a proven VEEV target *in vivo* (S2A and S2B Fig). Viral titers were unaltered between the two viruses in both cell types (Fig 6A). There was significantly less viral RNA present in VEEV CPD infected cells at 4 and 8 hpi, but no differences at later time points (S2C Fig). However, analysis of the amount of viral RNA in the cell culture media showed there was a significant decrease in extracellular viral RNA at later time points: 12 (Vero cells only), 16 and 24 hpi (Fig 6B and S2B Fig). An analysis of extracellular genomic copies to particle forming units (PFU) ratio was performed and the genomic copies to PFU ratio was determined to be lower in VEEV CPD vs. VEEV TC-83 (Fig 6C). These data suggest that cells infected with VEEV CPD output more functional viral particles than VEEV TC-83, potentially due to more efficient viral packaging and this phenotype is due to loss of capsid phosphorylation.

To determine the stability of the VEEV CPD mutant sites, we passaged VEEV CPD in Vero cells 10 times and purified six different plaques for viral sequencing. No sequence reversion at the mutated residues was observed in any of the six plaques tested. Sequencing of the entire capsid region revealed only one amino acid mutation in one plaque: Gly to Tyr in position 76. This residue is found within the SD3 domain of capsid which is involved in RNA binding and nucleocapsid assembly [27]. These results suggest that there is limited selective pressure for the phosphorylation sites to revert *in vitro* and are consistent with the unaltered replication kinetics of VEEV CPD.

### VEEV CPD binds RNA more efficiently than VEEV TC-83

**Fig 7:**
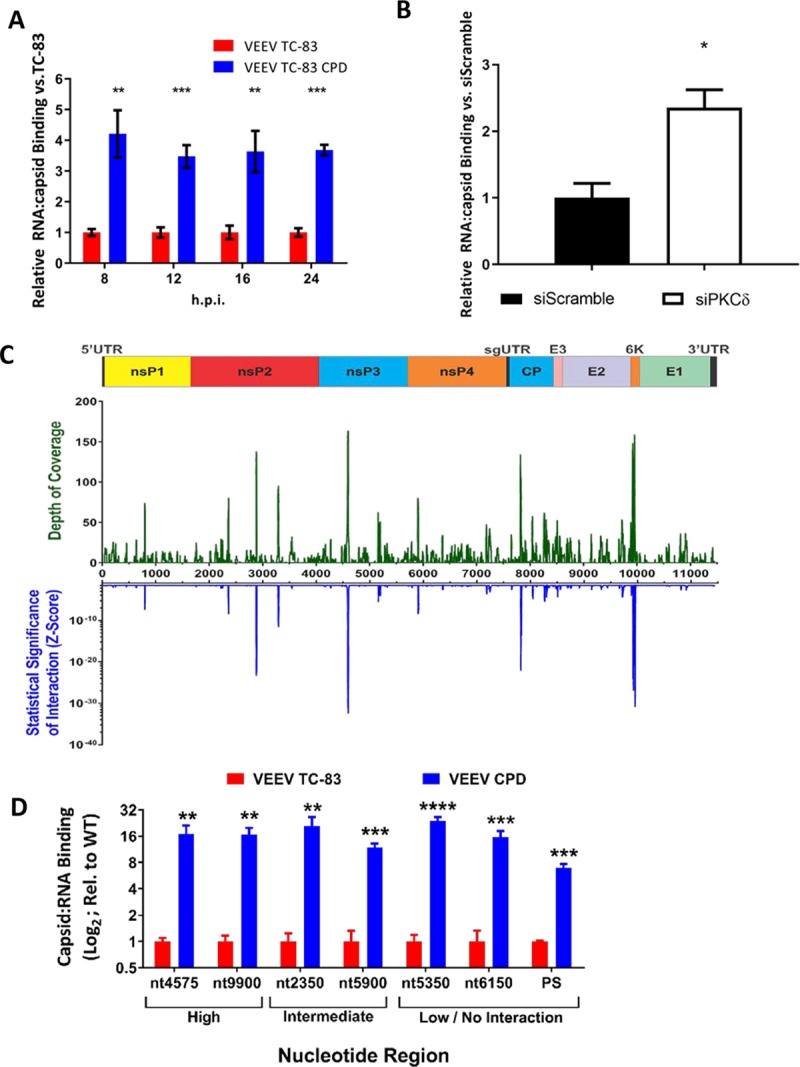
VEEV CPD binds RNA more efficiently than VEEV TC-83. A) Vero cells were infected with VEEV TC-83 or VEEV CPD (MOI 1.0). Cells were collected at the indicated time points, cross-linked, and cell lysates were immunoprecipitated with either α-HA or α-VEEV capsid antibodies. RT-qPCR against the VEEV packaging signal was performed on RNA isolated from immunocomplexes and normalized relative to a VEEV RNA standard curve generated during the same reaction. Genomic copies per reaction from α-HA immunoprecipitated samples were subtracted from the associated genomic copies from α-capsid immunoprecipitated samples to remove background and then the % RNA bound to capsid was determined by dividing the genomic copies from the input RNA by the normalized immunoprecipitated RNA. RNA:capsid binding of VEEV CPD was normalized relative to VEEV TC-83 samples. B) U87MG cells were transfected with 50 nM scramble control or PKCδ siRNAs. Seventy-two hours post-transfection, cells were infected with VEEV TC-83 (MOI 1.0). Samples were collected at 16hpi and processed in the same manner as panel A. C) A graphical representation of the VEEV Capsid CLIP-Seq data. On the Y-axes are the mean depths of coverage for the VEEV anti-Capsid data sets (Top) and relative statistical significance (Bottom) plotted with respect to nucleotide position (X-axis). Shown above the graphs is a schematic representation of the VEEV TC-83 reference genome (drawn to scale with the X-axis). D) Vero cells were infected with VEEV TC-83 or VEEV CPD (MOI 1.0). Cells were collected at 16 hpi, cross-linked, and cell lysates were immunoprecipitated with either α-HA or α-VEEV capsid antibodies. RT-qPCR against the VEEV canonical packaging signal and the sites identified in panel C was performed on RNA isolated from immunocomplexes and normalized relative to a VEEV RNA standard curve generated during the same reaction. Data analysis was performed as described in panel A. Data is average of 3 biological replicates. * = p<0.05, ** = p<0.005, *** = p<0.0005, **** = p<0.00005.

We have previously shown that inhibiting the dephosphorylation of capsid results in less viral RNA (vRNA) binding to capsid [24]. Given these results and that our capsid phosphorylation deficient virus displays a lower genomic copies to PFU ratio, we hypothesized that capsid phosphorylation mediates vRNA binding. In order to test this hypothesis, we investigated whether VEEV CPD had an effect on vRNA binding to capsid. Cells were infected with either VEEV TC-83 or VEEV CPD, fixed in paraformaldehyde, lysed, and immunoprecipitated for VEEV capsid. After immunoprecipitation, RNA was isolated and RT-qPCR was performed with primers targeting the canonical VEEV RNA packaging signal [28]. Results indicated that almost 4 times more viral RNA bound to capsid in the VEEV CPD vs. parental virus at all times post infection tested (Fig 7A). Loss of PKCδ through siRNA transfection also resulted in increased capsid viral RNA binding, further solidifying the link between VEEV CPD and PKCδ (Fig 7B).

We next wanted to understand how capsid phosphorylation globally affects viral RNA binding. Previous work with Sindbis virus (SINV) indicated that SINV capsid bound to multiple regions of viral RNA in the cytoplasm [29]. To this end, CLIP-seq studies were performed to determine if VEEV capsid interacts with viral RNA at sites other than the packaging signal. Briefly, 293HEK cells were infected with a GFP-reporter expressing strain of VEEV TC-83, and at 16 hpi the cells were washed and cross-linked via ultraviolet irradiation. After the generation of whole cell lysates, anti-capsid or nonspecific antibodies were used to immunoprecipitate RNA:Protein complexes. The RNA:Protein complexes were fragmented via RNAse treatment on the bead, and after extensive washing the immunoprecipitated RNA fragments were eluted via Proteinase K digestion and used as the starting material for cDNA library generation.

The CLIP-seq libraries were then aligned to the reference genome to reveal sites of VEEV Capsid:RNA interaction. CLIP-seq analysis of the cytoplasmic VEEV capsid:RNA interactions revealed distinct sites of significant enrichment, including an enriched cluster within the coding region of the GFP fluorophore (as shown in S3 Fig). The precise implications of this interaction, or its biological impact on viral infection are unknown; however, as the enrichment of this site could inadvertently mask the statistical significance of the interaction identification process by reducing the significance of all other viral RNA regions, and all downstream analyses utilized parental TC-83, we elected to censor the region from our downstream analyses. Thus, similarly to what was observed for SINV, distinct viral interaction sites were observed for the VEEV capsid protein and the vRNA (Fig 7C). Interestingly, no significant enrichment was observed at the region of the genome corresponding to the VEEV Packaging Signal (PS). However, it should be noted that a similar phenomenon was observed for SINV (27). Curiously, unlike SINV, VEEV capsid:RNA interactions were observed in both the nonstructural and structural coding regions. Comparative analysis of the molecular composition and structure for the SINV and VEEV Capsid:RNA interaction sites (including those of the GFP fluorophore) fails to identify any particular motif or structural element. Nonetheless; the lack of an identifiable canonical interaction motif may be entirely due to the limited number of interaction sites observed for each virus, rather than a differential set of binding proclivities.

To determine the impact of VEEV capsid phosphorylation on vRNA binding, we utilized quantitative RNA-IP experiments to probe the interaction between the capsid protein and the vRNA at sites identified as highly enriched, intermediately enriched, and non-enriched by our CLIP-seq studies. In addition to the Capsid:RNA interaction sites, we also assessed binding at the VEEV PS (26). As shown in Fig 7D, the VEEV CPD mutant had enhanced binding relative to parental TC-83 at all sites tested, including those with no significant enrichment as determined via CLIP-seq. Thus, these data suggest that phosphorylation of capsid is globally inhibitory to viral RNA binding.

### VEEV CPD virus is attenuated in a mouse model

**Fig 8:**
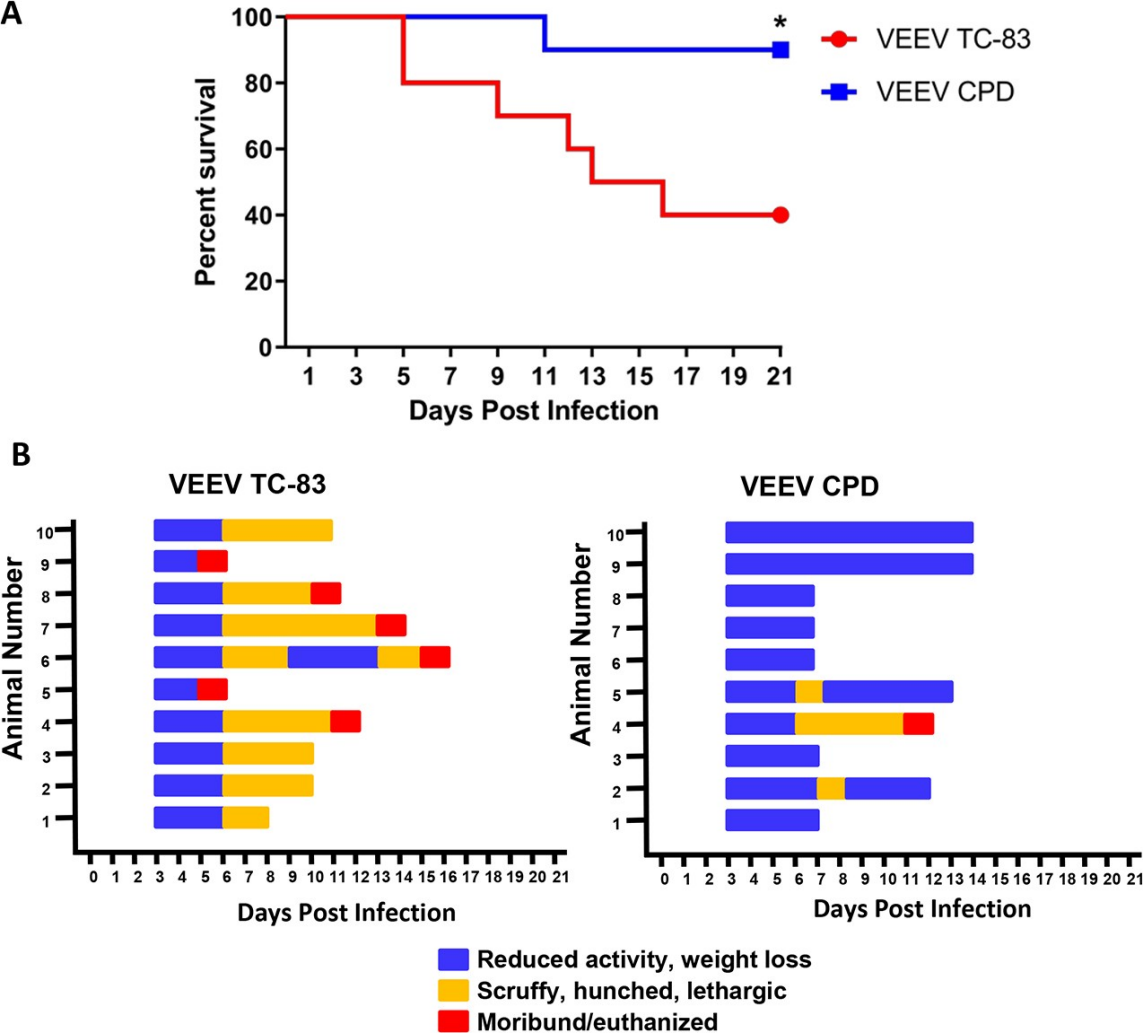
VEEV CPD is attenuated in mice. A) Kaplan-Meier survival plot of mice intranasally infected with 2 x 10^7^ pfu/mouse of either VEEV TC-83 or VEEV CPD. N = 10 per group. * = p<0.05. B) Mice were monitored at least daily for clinical symptoms of disease over 21 days. Data are plotted per animal per day. Blue lines indicate a score of 1–3 (primarily reduced activity and weight loss); Orange lines indicate a score of 4 or higher (primarily scruffy and hunched appearance, lethargy, severe weight loss); and red indicates the animal was moribund and euthanized or found dead upon observation.

Viral genomic copies to PFU ratios have been shown to be a determinant of viral attenuation. Ebola virus becomes attenuated in macaques as the genomic copies to PFU ratio decreases [30], whereas increased genomic copies to PFU ratio shows attenuation in Bunyamwera virus [31]. To determine if the mutations in VEEV capsid phosphorylation sites cause attenuation of VEEV TC-83 *in vivo*, six-week-old female C3H/HeN mice were challenged intranasally with a lethal dose of VEEV TC-83 or VEEV CPD. Mice were monitored for survival over 21 days and were observed daily for clinical symptoms of disease, weight loss, and body temperature. Sixty percent of mice infected with VEEV TC-83 succumbed to infection by 15 days post-infection while 90% of mice infected with VEEV CPD survived the infection (Fig 8A). Furthermore, survivors infected with VEEV CPD showed less severe clinical signs of infection compared to TC-83 infected mice (Fig 8B). Mice infected with TC-83 displayed mild signs of clinical illness (decreased activity, weight loss) by day 3 post-infection and increased in severity (scruffy, hunched, lethargic, significant weight loss) by day 5/6 post-infection. Surviving TC-83 mice recovered by day 11 (Fig 8A). Meanwhile, mice infected with CPD mutant virus displayed mild signs of clinical illness (decreased activity, weight loss) with only 3 of the mice increasing to more severe signs and one mouse succumbing to infection (Fig 8B and S4 Fig). These data suggest that VEEV CPD is attenuated in mice, warranting further study into its potential for a vaccine strategy. However, future studies identifying viral load and distribution of mice infected with VEEV CPD are required.

## Discussion

Our data demonstrated that PKCδ interacts with VEEV capsid, as indicated by its co-immunoprecipitation with capsid and co-localization by confocal microscopy during VEEV infection. PKC is a family of serine/threonine protein kinases that have been implicated in numerous cellular processes including transcriptional regulation, membrane structure modulation, immune mediation and cell growth regulation [32]. PKC has been implicated in several different viral life cycles. Inhibition of PKCα causes an increase in dengue virus replication, potentially by acting as a restricting mechanism that regulates dengue virus replication [33]. PKC is also involved in the lytic cycle switch of Epstein-Barr Virus by modulating microtubule depolymerization [34]. Additionally, Hepatitis B virus inhibits PKCδ causing decreased STAT activation during infection [35]. PKCδ, specifically, has been shown to be involved in HIV-1, SINV, and porcine reproductive and respiratory syndrome virus replication [36–38]. PKCδ is a novel isoform of the PKC family of kinases and plays conflicting roles in cell survival and cell death signaling. It is pro-survival and anti-apoptotic during cytokine receptor-initiated cell death, but it also can be a pro-apoptotic protein during DNA damage signaling [39,40]. In our study, we find that PKCδ regulates VEEV capsid binding to viral RNA.

Since our data indicated PKCδ is associated with VEEV capsid, we tested the importance of PKCδ during infection. A significant decrease of viral titers was observed starting at 8 hpi and continuing for at least 16 hpi after PKCδ knockdown by siRNA. To more specifically assess the importance of VEEV capsid phosphorylation, a VEEV capsid phosphodeficient mutant on a TC-83 backbone was constructed. Interestingly, the phospho-deficient mutant virus, VEEV CPD, displayed no significant difference in titer when compared to the parental TC-83. These data are contrary to the siRNA data which show a decrease in viral titer after knockdown of PKCδ. These data could indicate that PKCδ is important in more than one aspect of the viral life cycle and that knockdown of PKCδ prevents another unknown mechanism of viral replication from functioning properly. It is also possible the phosphorylation of individual residues plays independent roles and PKCδ is only impacting one or some of the phosphorylated residues. Future studies with viruses containing single mutations and/or double and triple mutant combinations will be necessary to determine the importance of individual phosphorylation sites.

**Fig 9:**
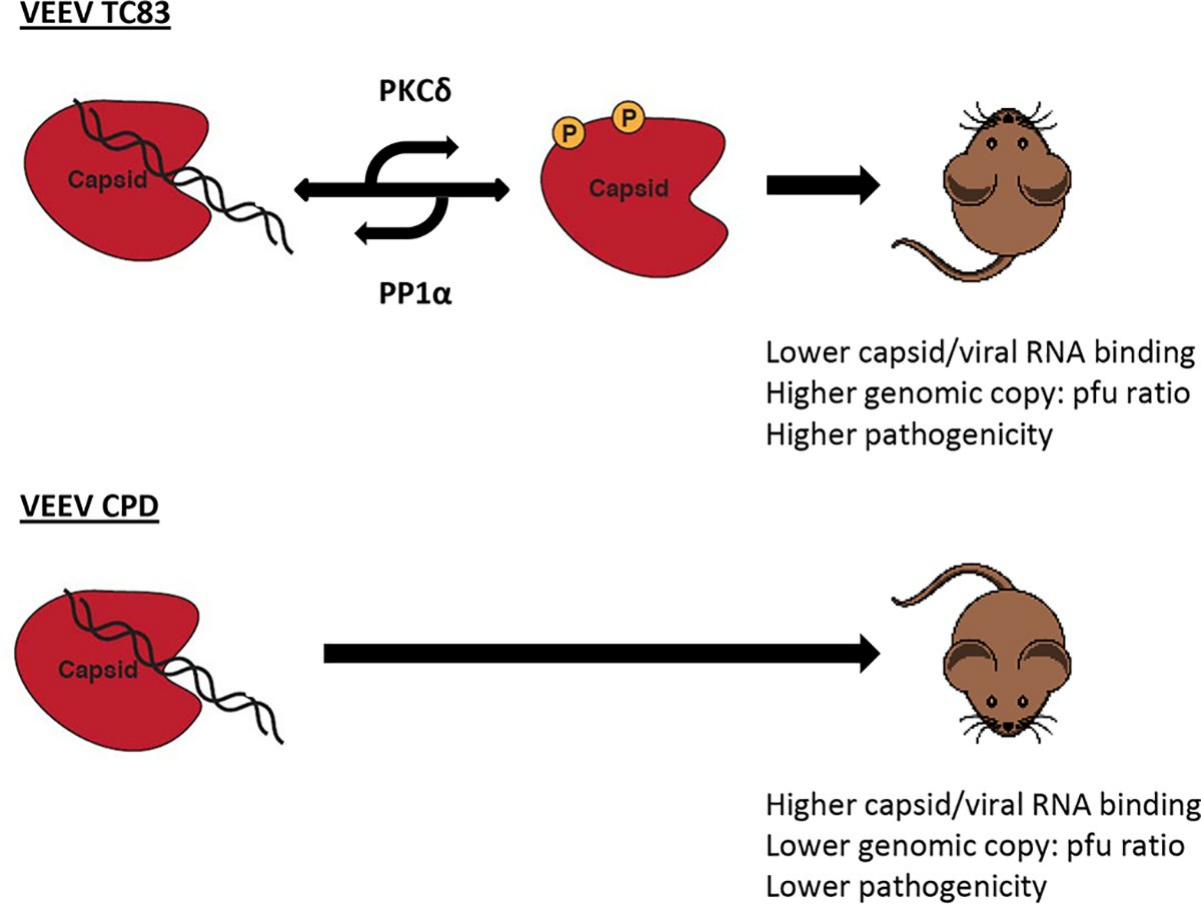
Working model of the capsid phosphorylation and its impact on viral pathogenesis. Our working model is that capsid phosphorylation is a mechanism important for regulating capsid:viral RNA binding. Capsid is dephosphorylated by PP1α increasing its ability to bind to viral RNA and conversely phosphorylation by PKCδ decreasing viral RNA binding. Capsid phosphorylation regulates VEEV’s genomic copy to pfu ratio and viral pathogenesis.

Our results show that knockdown of PKCδ and mutation of capsid phosphorylated residues decreases the level of phosphorylation on VEEV capsid to almost undetectable levels. We identified the phosphatase responsible for capsid dephosphorylation, PP1α, in our previous study [24], but no kinase had been identified up to that point. The knockdown of PKCδ coupled with the drop in capsid phosphorylation strongly suggests that PKCδ is the kinase responsible for phosphorylating capsid. Our previous data suggest that higher levels of capsid phosphorylation results in less viral RNA binding and it has been shown in the related *Togavirus*, Rubella virus, that phosphorylation of capsid protein regulates its RNA binding and viral replication [41]. We compared the amount of RNA bound to capsid in either VEEV TC-83 or VEEV CPD and our results indicated that there is more viral RNA bound to capsid in the mutant strain. Coupling these data with our previous study that shows that higher levels of phosphorylation causes less viral RNA bound to capsid, we propose that capsid phosphorylation/dephosphorylation is a cycle that regulates RNA binding (Fig 9). Interestingly, VEEV CPD also displayed a lower genomic copy to PFU ratio and decreased pathogenicity. As VEEV CPD replicated to similar levels as parental VEEV TC83 *in vitro*, these data suggest that capsid phosphorylation is not necessary for viral replication, but rather regulates viral pathogenesis. The altered pathogenesis may be linked to VEEV CPD producing less non-infectious particles. Multiple viruses have been shown to produce non-infectious particles, including influenza virus [42,43], hepatitis B virus [44], herpes simplex virus [45], and yellow fever virus [46]. The non-infectious particles can be immature viruses, defective interfering particles, or semi-infectious particles with varying roles in pathogenesis ranging from increasing disease, stimulating the innate immune response, serving as a decoy for antibodies, or facilitating recombination [47–49]. Our data support a model whereby capsid phosphorylation facilitates the release of non-infectious particles which aid in disease development. Future studies will be aimed at determining how capsid phosphorylation regulates pathogenesis.

PKCδ has previously been shown to phosphorylate the capsid protein of Rubella virus which regulates binding of viral RNA to capsid [41] however, our study is the first to put forth a mechanism for any alphavirus capsid phosphorylation cycle. While PKCδ is at least one kinase that regulates VEEV capsid phosphorylation, there could potentially be other kinases that are important during the life cycle of VEEV such as DNAPK and PKA, as predicted *in silico*. PKC was also predicted to phosphorylate capsid proteins of EEEV and WEEV, but loss of PKC through siRNA transfection had no impact on viral replication. However, there were multiple residues of EEEV and WEEV capsid proteins that are predicted to be phosphorylated and by alternative candidate kinases such as PKA. This is an area of importance for future study as phosphorylation of alphavirus capsid proteins may be used as a conserved mechanism to regulate capsid functionality.

Cellular and viral protein phosphorylation is critical during the life cycle of several viruses [50–53]. Furthermore, elucidating the mechanism of phosphorylation during infection and the purpose it serves can help lead to attenuation of viruses for potential vaccine candidates. Several viruses can be attenuated through mutation of their phosphorylation sites including T215 on the NS1 protein of Influenza A virus [54] as well as S86 and S151 on the measles virus phosphoprotein [55]. Our data add to the list of viruses attenuated by ablating an important phosphorylation event. It shows that mutating the phosphorylation sites on VEEV capsid attenuates the virus *in vivo*. Additionally, surviving mice infected with VEEV CPD display only mild clinical symptoms of infection suggesting that VEEV CPD does not induce the pathogenesis observed with VEEV TC-83. Further studies are being performed in our lab to determine viral kinetics *in vivo* and if VEEV CPD at a lower dose can potentially eliminate clinical symptoms observed from this study and also protect against lethal challenge with aerosolized VEEV TrD.

## Materials and methods

### Viral infection

VEEV TC-83 and VEEV Trinidad Donkey (TrD) were obtained from BEI Resources. WEEV 1930 California was purchased from ATCC (Manassas, VA). EEEV GA97 was kindly provided by Jonathan Jacobs of MRIGlobal [56]. VEEV TC-83 V5-6K/TF was made through standard molecular biology techniques with the V5 tag placed in the N-terminus between the first and second amino acid of the 6K/TF protein. The location of the tag was determined by comparing the location of tags placed in other alphaviruses [57,58]. All experiments with VEEV TrD, EEEV, and WEEV were performed under BSL3 conditions whereas experiments with VEEV TC-83 were performed under BSL2 conditions. All work involving select agents was registered with the Centers for Disease Control and Prevention and conducted at George Mason University’s Biomedical Research Laboratory, which is registered in accordance with federal select agent regulations.

For infections, virus was added to supplemented Dulbecco’s Modified Eagles Medium (DMEM) to achieve an MOI (relative to cell type used) of either 0.1 or 1. Cells were infected for one hour at 37°C and rocked every 15–20 min. Cells were then washed with sterile PBS pH 7.4 and media was added. Crystal violet plaque assays were performed in Vero cells (ATCC, CCL-81) to determine viral titers as previously described [59].

### Cell culture

Vero, HEK293T (ATCC, CRL-3216) and U87MG (ATCC, HTB-14) cells were maintained at 37°C, 5% CO2 in Dulbecco’s modified Eagle medium (DMEM) supplemented with 10% fetal bovine serum (FBS), 1% glutamine, and 1% penicillin/streptomycin. BHK-21 (ATCC, CCL-10) cells were maintained at 37°C, 5% CO2 in Modified Eagle Medium (MEM) supplemented with 10% fetal bovine serum (FBS), 1% glutamine, and 1% penicillin/streptomycin. Human Primary Astrocytes (Lonza CC-2565) were maintained at 37°C, 5% CO2 in Astrocyte Growth Medium Bullet Kit (Lonza CC-3186).

### Immunoprecipitation and Western blot analysis

Protein lysates were obtained using lysis buffer consisting of 50mM Tris-HCl pH7.4, 120mM NaCl, 5mM EDTA, 0.5% NP-40, 50mM NaF, 0.2mM Na_3_VO_4_, cOmplete Protease Inhibitor Cocktail (Sigma-Aldrich, 11697498001). Following cell lysis, 1 mg of total protein per sample was immunoprecipitated with 1 μg of anti-PKCδ (Cell Signaling, 9616) rabbit primary antibody, Anti-VEEV-capsid (BEI Resources, NR-9403) goat primary antibody, or anti-HA (Cell Signaling, 3724) rabbit primary antibody at 4°C overnight. Following immunoprecipitation, antibody:antigen complexes were bound to Protein G Dynabeads (Thermo Fisher, 10004D). Beads containing the protein complexes were washed one time in TNE_600_ with 0.1% NP-40, twice in TNE_150_ with 0.1% NP-40, and once in PBS. Western blot loading buffer, consisting of Novex Tris-Glycine Sample Loading Buffer SDS (Thermo Fisher, LC2676), T-PER Tissue Protein Extraction Reagent (Thermo Fisher, 78510), EDTA, cOmplete Protease Inhibitor Cocktail, 50mM NaF, 0.2mM Na_3_VO_4_, and 300 mM DTT, was added to antibody:dynabead complexes and samples were run on NuPAGE 4–12% Bis-Tris Protein Gels (Thermo Fisher, NP0321). Following transfer to a PVDF membrane, membranes were blocked in either 5% BSA-TBS-Tween or 3% Milk-PBS-Tween and incubated in the indicated primary antibody [anti-PKCδ (Cell Signaling, 9616) 1:1000 dilution), anti-PKCμ (Cell Signaling, 90039, 1:1000 dilution), anti-PKCα (Cell Signaling, 2056, 1:1000 dilution), anti-PKCζ (Cell Signaling, 9368, 1:1000 dilution), anti-VEEV-capsid (BEI Resources, 1:1000 dilution), anti-phosphoserine (Millipore, AB1603, 1:500 dilution), anti-phosphothreonine (Millipore, AB1607, 1:500 dilution), anti-STAT3 (Cell Signaling D3Z2G, 1:1000 ratio) or anti- V5 (BioRad MCA 1360, 1:1000 dilution)] overnight at 4°C. Following primary antibody incubation, membranes were washed and incubated for 1 hour in the appropriate secondary antibody, either anti-rabbit HRP-conjugated (Cell Signaling, 7074), anti-mouse HRP-conjugated (Cell Signaling, 7076) or anti-goat HRP-conjugated. Membranes were imaged on a Chemidoc XRS molecular imager (BioRad) using the SuperSignal West Femto Maximum Sensitivity Substrate kit (Thermo Fisher, 34095). Non-immunoprecipitated samples were processed in the same way without performing the immunoprecipitation steps.

### CLIP-Seq analysis of VEEV capsid binding

The identification of cytoplasmic VEEV Capsid:RNA interactions was conducted as described in [29]), with minor differences. Specifically, 2x10^7^ 293HEK cells were infected with VEEV TC-83.dsGFP at an MOI of 5 PFU/cell, and after a 16 hour incubation period the cells were washed and irradiated with 5700x100 μJoules per square centimeter. After crosslinking whole cell lysates were generated via the addition of RIPA buffer (50mM Tris [pH 7.6], 150mM NaCl, 1.0% NP-40, 0.5% Sodium Deoxycholate, 0.1% SDS). The whole cell lysate was solubilized via repeated passage through a 30-gauge needle, and insoluble materials were removed from the lysate via centrifugation at 16,000xg for 5 minutes at 4°C. The clarified lysates (~500μl per reaction) were then incubated with 50μl packed volume of Protein G Sepharose beads (Pierce) for 15 minutes at 4°C. The resin was removed by gentle centrifugation at 5,000xg for 5 minutes, and the pre-blocked lysates were transferred to fresh microfuge tubes containing polyclonal anti-Capsid or nonspecific IgG. After 2 hours of incubation at 4°C on a rotating mixer, 100μl of Protein G Sepharose was added to each tube and the incubation proceeded as before for another 2 hours. After the formation of the immunoprecipitation complex, RNAse A was added to each tube and the immunoprecipitations were incubated for 15 minutes at room temperature to fragment the bound RNA:Protein complexes. Then the nuclease treated resin was collected via centrifugation at 5,000xg for 2 minutes and washed three with excess volumes of RIPA buffer. After the removal of the contaminating RNAse and RNA fragments, the bound resin was washed twice more with 1xPBS, and the RNA fragments were eluted from the resin by way of Proteinase K digestion. The purified fragments were extracted using TRIzol and precipitated prior to the generation of cDNA libraries using the NEBNext Small RNA Sequencing (NEB) kit, as per the manufacturer’s instructions. The resulting libraries were sequenced on a HiSeq platform (Illumina).

The resulting cDNA library data was trimmed to remove indices and adaptors and trimmed for quality (>20 over a 4nt scanning window) and length (>10nt). The resulting cDNA data was aligned to the VEEV TC-83.dsGFP reference genome using Bowtie2 with the default parameters. Only the reads which aligned to the positive-sense RNA genome were mapped, and sequence coverage was clustered at a nucleotide level of resolution. The analysis of the clustering data was performed identically to that described in [29]. The sequencing data and their analyses as relevant to the studies described herein are available in the supplemental files accompanying this manuscript as S1 Data. In addition, the entire RNA sequencing data set has been deposited in the National Center for Biotechnology Information (NCBI) Gene Expression Omnibus, and can be accessed by the following URL: https://urldefense.proofpoint.com/v2/url?u=https-3A__www.ncbi.nlm.nih.gov_geo_query_acc.cgi-3Facc-3DGSE132125&d=DwIBAg&c=OAG1LQNACBDguGvBeNj18Swhr9TMTjS-x4O_KuapPgY&r=HAbXqcvDsSEpsfQ7-8oxF9_XjrPTRA0maMiRyITXKtM&m=mn_ESwz7wg_rIgqHybbAzs0w2PxZfCkymQnJpoVc3Tk&s=zwhEXJsjw_vFKC4S3I6Li0BN9V1KI7IUXIIaYN53S88&e=

To enhance the clarity of data presentation and enable congruence between the nucleotide positions and the native TC-83 sequence, we have censored the region corresponding to the GFP reporter during the presentation of the data. Nonetheless, the data deposited to the NCBI consists of the data set in its entirety, and the presentation of the uncensored alignment data may be found in S3 Fig.

### RNA immunoprecipitation

Infected cells were collected, washed in PBS, and cross-linked with 1% paraformaldehyde. Cross-linked cells were lysed in RIPA lysis buffer (50mM Tris-HCl, pH 7.5, 1% NP-40, 0.5% Sodium Deoxycholate, 0.05% Sodium Dodecyl Sulfate, 1mM EDTA, cOmplete Protease Inhibitor Cocktail tablet, and 150 mM NaCl) and ultrasonicated. Lysates were clarified by centrifugation and immunoprecipitated as described above with α-HA and α-VEEV capsid antibodies. Following immunoprecipitation, antibody:antigen complexes were bound to protein G Dynabeads as described above. Beads bound to antibody complexes were washed in high stringency RIPA buffer (50mM Tris-HCl, pH 7.5, 1% NP-40, 1% Sodium Deoxycholate, 0.1% Sodium Dodecyl Sulfate, 1mM EDTA, 1M NaCl, 1M Urea, and cOmplete Protease Inhibitor Cocktail tablet) and resuspended in TEDS buffer (50mM Tris-HCl, pH 7.0), 1% Sodium Dodecyl Sulfate, 5mM EDTA, and 10mM DTT). Samples were then processed for RNA extraction.

### RNA Isolation and RT-qPCR

Supernatants were collected to analyze extracellular viral RNA, and infected cells were lysed and collected in TRIzol (Thermo Fisher, 15596026) to analyze intracellular RNA. Extracellular viral RNA was isolated using Ambion's MagMax Viral RNA Isolation Kit (Thermo Fisher, AM1836) while intracellular RNA and immunoprecipitated RNA (and input RNA) was isolated using the Direct-zol RNA Miniprep Kit (Zymo Research, R2050) by following the manufacturer’s instructions. RT-qPCR was performed as described previously [60] for viral RNA using the Integrated DNA Technologies primer pairs (Table 2) and Taq-Man probe (6-carboxyfluorescein-TCGTCCGCACTGACATCTGTTGC-carboxytetramethylrhodamine) against the viral RNA packaging signal (nt 1057–1154) (26) or by SYBR Green for non-probe based assays. The absolute quantification was determined using StepOne Software v2.3 based on the threshold cycle relative to the standard curve. The standard curve was determined using serial dilutions of VEEV-TC-83 RNA at known concentrations. Relative RNA:capsid binding was determined by subtracting the background RNA bound to antibody and dynabeads (α-HA IP samples) from the experimental samples and compared to input RNA levels. Results were normalized to TC-83 for comparison.

**Table 2:** Primers used.

Primer Name	Sequence
2342 Fwd	CAGAACTGTGGACTCAGTGCT
2342 Rev	ACGGGGTGTTTGCATC
4569 Fwd	CTGGTGAGGGTGCATCCGA
4569 Rev	GCCCTTCCTTCCAGCCAAA
5348 Fwd	AACGTCAGCCGAGAC
5348 Rev	GCGAAGTAAGAGTTA
5886 Fwd	AGTCCAGGAAGG
5886 Rev	CTTTCATGTTCTCCA
6140 Fwd	CTATTTGGACATGGTTGACG
6140 Rev	AACTGGCAGTGTCTAAGCAGC
9920 Fwd	ATCGTAGTGACTCGCCTGCT
9920 Rev	GACACAGCACACGCACCT
Packaging Signal Fwd	GTGTGACCAAATGACTG
Packaging Signal Rev	ACCGTTGACGACTATAC

### Cell viability assays

For cell viability assays, cells were cultured as described above and transfected with siRNA at a final concentration of 50nM. Cells were incubated for 72 hours and ATP production was measured as an indication of cell viability using Promega’s Cell Titer-Glo (Promega, G7570). Assays were performed in white-walled, 96-well plates (Corning, 3610) seeded with 10,000 cells per well following the manufacturer’s protocol. Luminescence was detected on a Beckman Coulter DTX880 plate reader after 100ms integration per well.

### Transfections

For siRNA transfections, U87MG cells were transfected with DharmaFECT transfection reagent (GE Lifesciences, T-2001) for 72 hours prior to VEEV infection. For siRNA either On-Target Plus SMARTpool for PRKCD (GE Lifesciences, L-003524-00) or All Stars siRNA (Qiagen, SI03650318) negative control were used. For experiments with plasmids, we previously described a VEEV structural polyprotein construct [24] that was transfected using polyethylenimine (PEI) at a ratio of 4 μg PEI: 1 μg DNA.

### Confocal microscopy

Vero cells were grown on poly-L-lysine coated cover-slips in a 6-well plate, infected with VEEV TC-83 or mock-infected, and processed for immunofluorescence analysis as previously described [55]. Anti-VEEV-capsid goat primary antibody (1:1000 dilution) and Alexa Fluor 568 donkey anti-goat secondary antibody (Thermo Fisher, A11057, 1:500 dilution) were used to probe for capsid. Anti-PKCδ rabbit primary antibody (1:1000 dilution) and Alexa Fluor 488 donkey anti-rabbit secondary antibody (Thermo Fisher, A21206, 1:500 dilution) were used to probe for PKCδ. Anti VEEV-E2 mouse primary antibody (1:1000 dilution) and Alexa Fluor 568 anti-mouse secondary antibody (Thermo Fisher, A10037, 1:500 dilution) were used to probe for E2. Slides were imaged using an oil-immersion 60X objective lens on a Nikon Eclipse TE 2000-U confocal microscope, with all samples subjected to four line averaging. At least four images were taken of each sample, with one representative image shown. Each image was processed using Nikon NIS-Elements AR Analysis 3.2 software. Z-stack analysis was performed on Nikon NIS-Elements AR Analysis 3.2 software using the co-localization function. Briefly, the red (x-axis) and green (y-axis) channels were selected for comparison and the resulting scatter plot shows the intensity of each pixel within the image. The gate function was used to highlight the pixels that were the most intense for both channels. Pixels within the gates were analyzed for Pearson’s correlation.

### VEEV CPD mutant virus production

The original plasmid containing the infectious cDNA of VEEV TC-83 was obtained from Ilya Frolov at the University of Alabama Birmingham and described elsewhere [61]. Viral genome-coding cDNA was cloned under the control of the SP6 promoter. Mutations in the capsid-coding sequence of the VEEV TC-83 that prevents phosphorylation of the threonine and serine in positions 93, 108, 124 and 127 were introduced using standard DNA techniques. Briefly, in the plasmid that encodes VEEV TC-83 cDNA, the fragment of the capsid sequence between AflII and Bsu361 restriction sites was replaced by *in vitro* synthesized gBlock gene fragment (Integrated DNA Technologies). In the gBlock fragment, all the serine and threonine codons corresponding to the capsid amino acids in positions 93, 108, 124 and 127 were changed to alanine codons. Presence of the mutations in the phospho-deficient capsid mutant virus was confirmed by sequencing of several viral genomes collected from infected cells. All of the sequences and details of the cloning procedures can be obtained upon request. *In vitro* transcription was performed on the mutated TC-83 molecular clone using the MEGAscript SP6 Transcription Kit (Thermo Fisher, AM1330). Resultant RNA was electroporated into BHK cells and virus was collected 24 hours post electroporation. Passage 0 virus titer was determined by plaque assay as described above and used to infect Vero cells to grow virus for passage 1 as described above. Passage 1 virus was used in subsequent experiments.

### Animal experiments

Six-week-old female C3H/HeN mice were obtained from Charles River Laboratories. Groups of 10 mice were individually identified via tattoo and had temperature transponders (BioMedic Data Systems) implanted subcutaneously 3 days prior to the start of the study. Mice were infected intranasally with a dose of 2 x 10^7^ pfu/mouse of VEEV TC-83 or VEEV CPD. Animals were observed for survival over the course of 21 days. Mice were observed daily for signs of clinical illness as determined by our clinical sign scoring sheet developed for TC-83 animal studies. Mice were scored individually on the following parameters: appearance, mobility, attitude, and body condition. Appearance was scored as follows: 0- smooth coat, bright eyes; 1- slightly scruffy and/or hunched at rest; 2- scruffy and/or hunched at rest; 3- very scruffy and/or hunched at rest, mild eye crust; and 4- very scruffy and/or hunched at rest, closed inset eyes. Mobility was scored as follows: 0- active, exploring cage; 1- less active, walking; 2- slow movement; 3- no movement; and 4- unresponsive. Attitude was scored as follows: 0- alert, 1- mildly lethargic, 2- lethargic, and 3- unaware. Body condition was scored as follows: 0- normal or overweight; 1- underconditioned; and 2- emaciated. Scores for each parameter were summed for a total score and mice scoring 0–5 were observed once daily, mice scoring 6–10 were observed twice daily, and mice scoring an 11 or greater were humanely euthanized. Measurements of well-being, weights and body temperatures were recorded each day. Personnel performing clinical observations, weights and body temperatures were blinded to the animal groups. Experiments were performed in animal biosafety level 2 (ABSL-2) laboratories in accordance with the National Research Council’s *Guide for the Care and Use of Laboratory Animals* (57) and under George Mason University IACUC protocol number 0384.

### Statistical analysis

Statistical analysis was performed using GraphPad Prism software (http://www.graphpad.com). All experiments were done with at least 3 biological replicates and statistical significance was evaluated using Student‘s T-test. P-values are indicated within the figure by an asterisk where * = p<0.05, ** = p<0.005, *** = p<0.0005, and **** = p<0.0001.

### Ethics statement

All animal procedures were carried out under approval of the Institutional Animal Care and Use Committee of George Mason University under protocol #0384. Animal care and use were performed in accordance with the recommendations in the Guide for the Care and Use of Laboratory Animals of the National Research Council.

## Supporting information

S1 Fig PKCδ coimmunoprecipitates with VEEV capsid and STAT3 but not VEEV TF/6K and PKCδ knockdown impacts STAT3 phosphorylation. A) Vero cells were mock-infected or infected with VEEV TC-83 V5-6K/TF (MOI of 1.0) for 18 hours. Cells were lysed and 1 μg of α-PKCδ antibody was added to 1 mg of protein lysate. Protein complexes were bound to Protein G Dynabeads, and samples were run on SDS-PAGE and western blot analysis was performed for PKCδ, VEEV capsid, STAT3, and VEEV 6K/TF (V5 tag). B) U87MG cells were transfected with 50 nM scramble control or PKCδ siRNAs. Seventy-two hours post-transfection, cells were infected with VEEV TC-83 (MOI 0.1) and cell lysates collected. Western blot analysis was performed with anti-PKCδ, anti-STAT3 (Ser727), and anti-actin antibodies.
(TIF)

S2 Fig VEEV CPD is more infectious in Human Primary Astrocytes and intracellular VEEV RNA is decreased at early time points in cells infected with VEEV CPD vs. VEEV TC-83. Vero cells were infected with either VEEV TC-83 or VEEV CPD (MOI 0.1) and viral supernatants collected at the indicated time points for viral titer determination via A) plaque assay or B) RT-qPCR. C) Vero cells were infected with VEEV TC-83 or VEEV CPD at an MOI of 0.1. RNA was extracted from cells at the indicated time points and RT-qPCR was performed. Values are an average of 3 biological replicates. * = p<0.05, ** = p<0.01.
(TIF)

S3 Fig Alignment of the CLIP-seq data set to the TC-83 dsGFP Reference genome. Data identical to that presented in Fig 7C, with the exception that the region corresponding to the GFP coding region is represented within the figure.
(TIF)

S4 Fig Average Body temperature and weights of mice infected with VEEV TC-83 or VEEV CPD. A) Daily average body temperature readings from mice infected with VEEV TC-83 or VEEV CPD. B) Daily average body weights from mice infected with VEEV TC-83 or VEEV CPD.
(TIF)

S1 Dataset Files pertaining to the CLIP-Seq analysis of the VEEV Capsid:RNA interactions. Included are the parsed data sets obtained from alignment and their analyses. The statistical analysis of the raw read data can be found in S2 Data.
(XLSX)

S2 Dataset Data pertaining to the CLIP-Seq analysis of the VEEV Capsid:RNA interactions. Included are statistical analyses of the raw read data. The parsed data sets can be found in S1 Data.
(XLSX)
