# Supplementary figures and images for: Protein Kinase C subtype δ interacts with Venezuelan equine encephalitis virus capsid protein and regulates viral RNA binding through modulation of capsid phosphorylation
Source: PLoS Pathog. 2020 Mar 9;16(3):e1008282. doi: 10.1371/journal.ppat.1008282 (PMC7082041; doi:10.1371/journal.ppat.1008282)

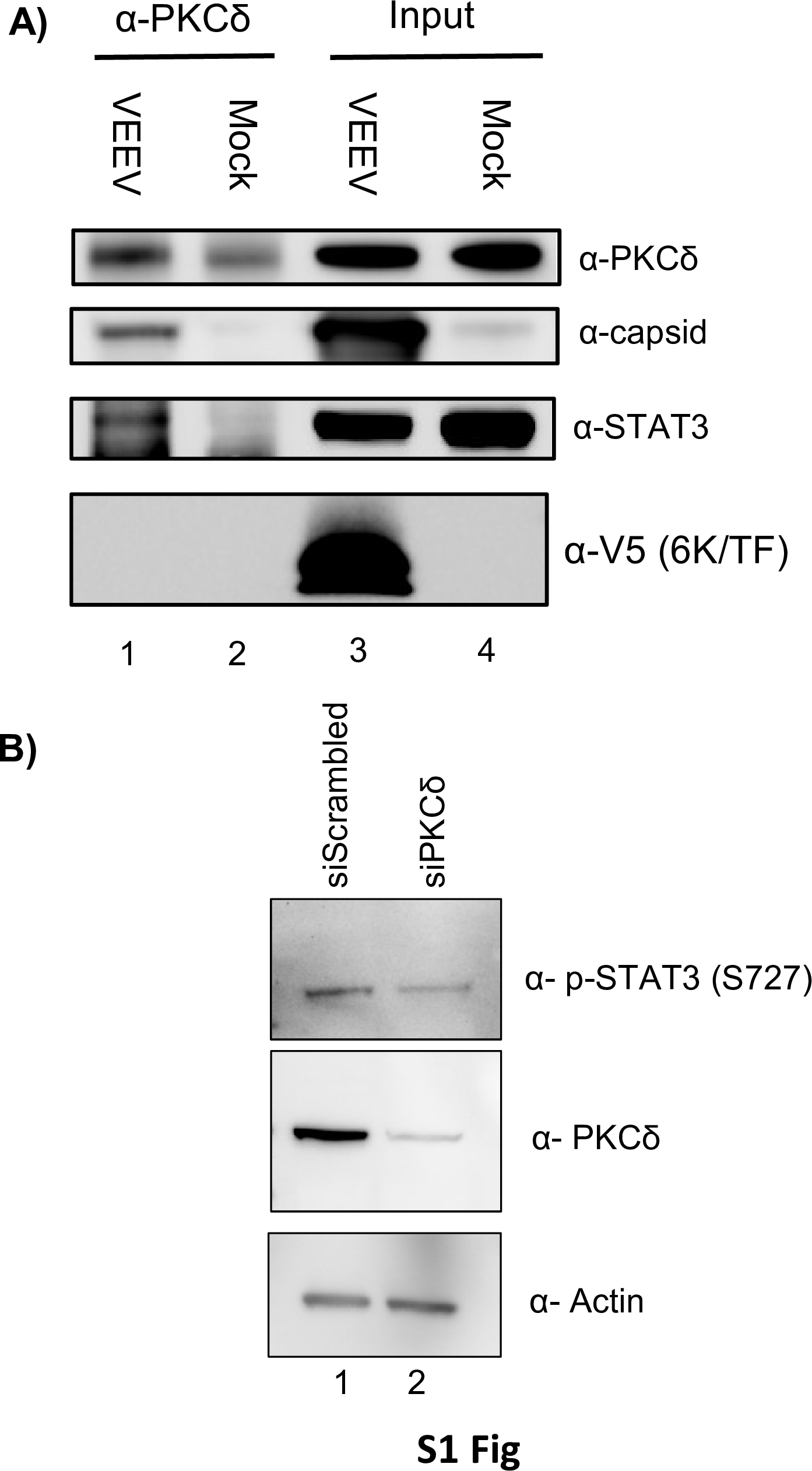

Supplement: S1 Fig — A) Vero cells were mock-infected or infected with VEEV TC-83 V5-6K/TF (MOI of 1.0) for 18 hours. Cells were lysed and 1 μg of α-PKCδ antibody was added to 1 mg of protein lysate. Protein complexes were bound to Protein G Dynabeads, and samples were run on SDS-PAGE and western blot analysis was performed for PKCδ, VEEV capsid, STAT3, and VEEV 6K/TF (V5 tag). B) U87MG cells were transfected with 50 nM scramble control or PKCδ siRNAs. Seventy-two hours post-transfection, cells were infected with VEEV TC-83 (MOI 0.1) and cell lysates collected. Western blot analysis was performed with anti-PKCδ, anti-STAT3 (Ser727), and anti-actin antibodies. (TIF) [file ppat.1008282.s001.tif]

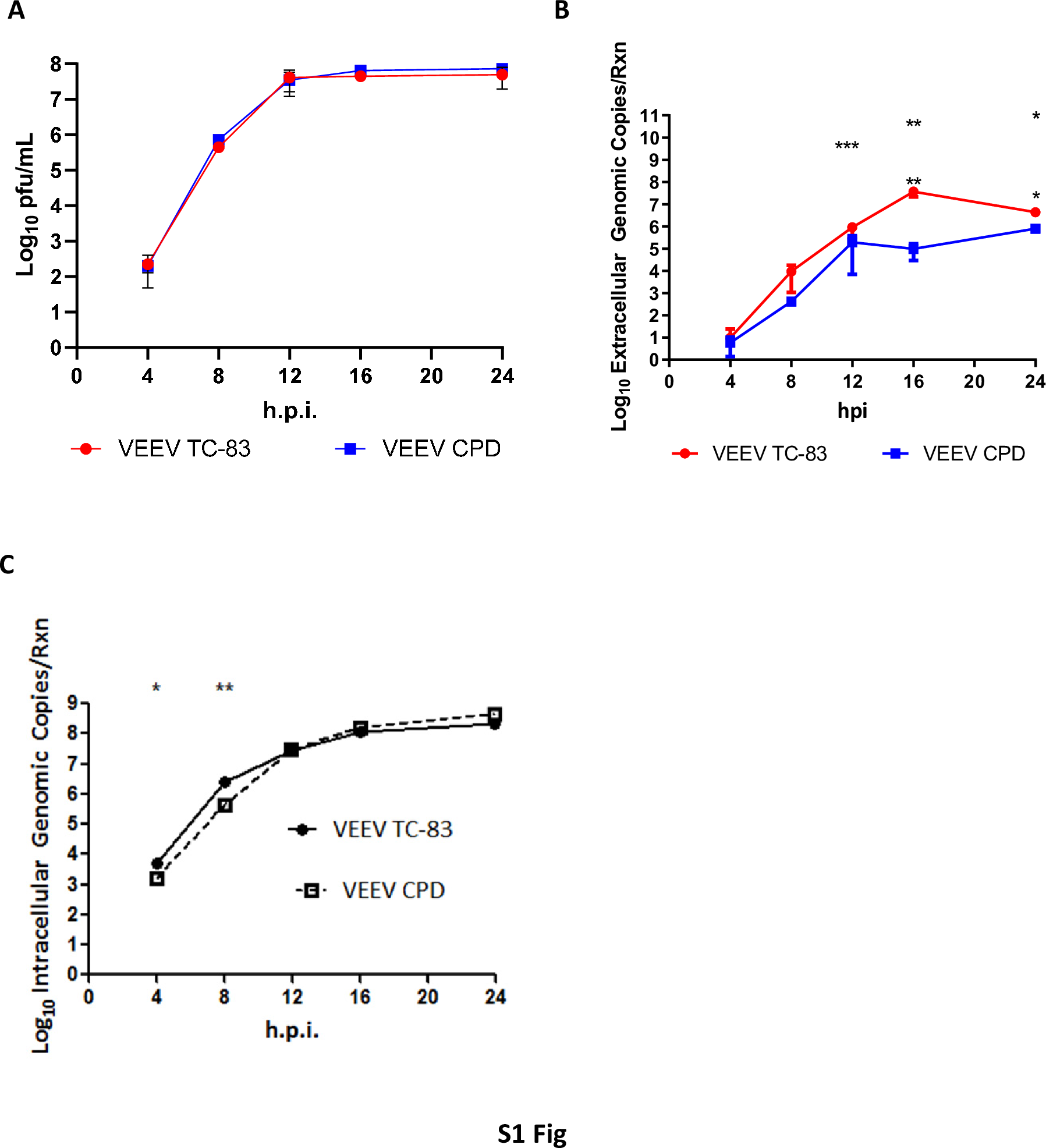

Supplement: S2 Fig — Vero cells were infected with either VEEV TC-83 or VEEV CPD (MOI 0.1) and viral supernatants collected at the indicated time points for viral titer determination via A) plaque assay or B) RT-qPCR. C) Vero cells were infected with VEEV TC-83 or VEEV CPD at an MOI of 0.1. RNA was extracted from cells at the indicated time points and RT-qPCR was performed. Values are an average of 3 biological replicates. * = p<0.05, ** = p<0.01. (TIF) [file ppat.1008282.s002.tif]

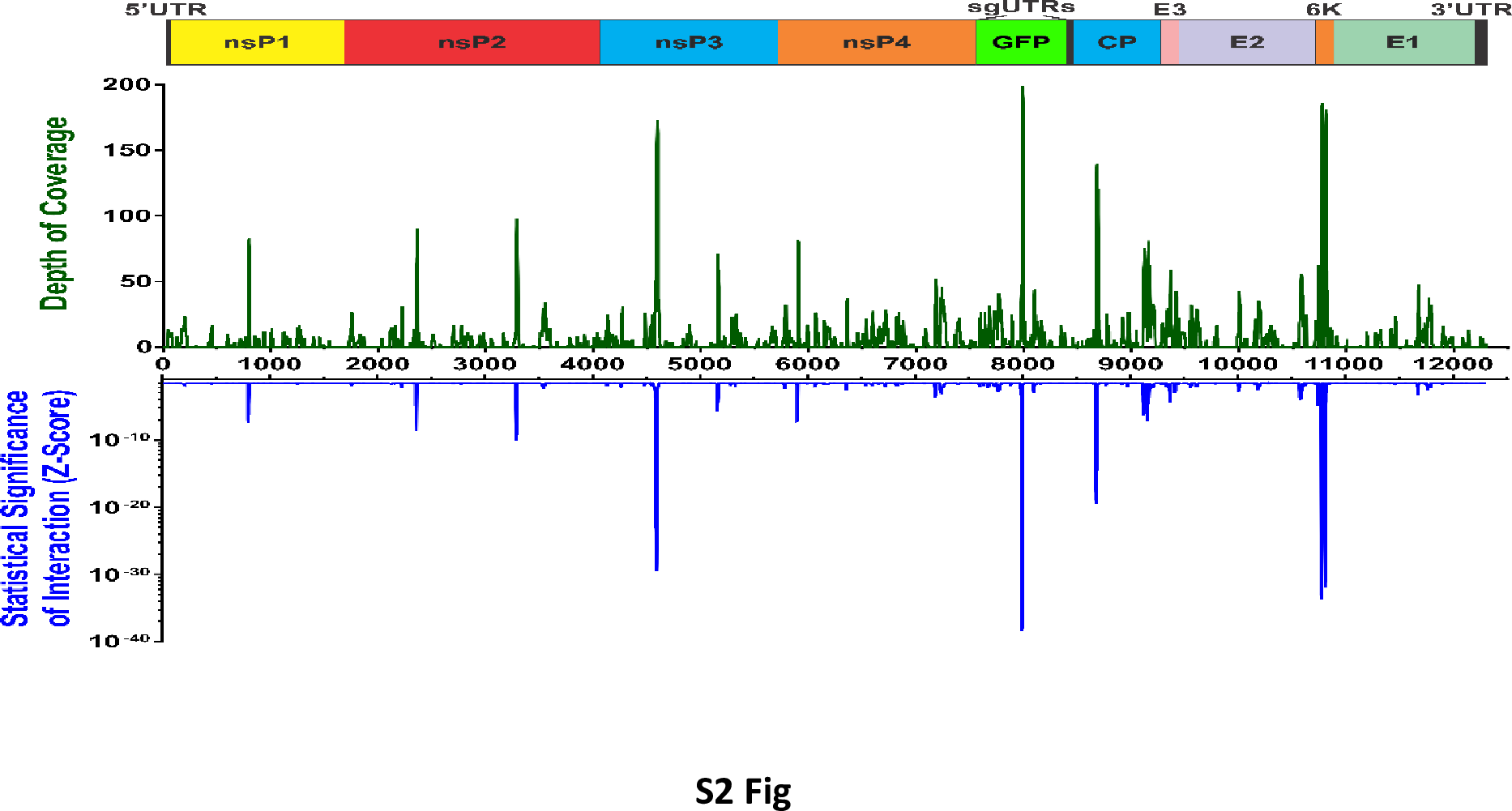

Supplement: S3 Fig — Data identical to that presented in Fig 7C, with the exception that the region corresponding to the GFP coding region is represented within the figure. (TIF) [file ppat.1008282.s003.tif]

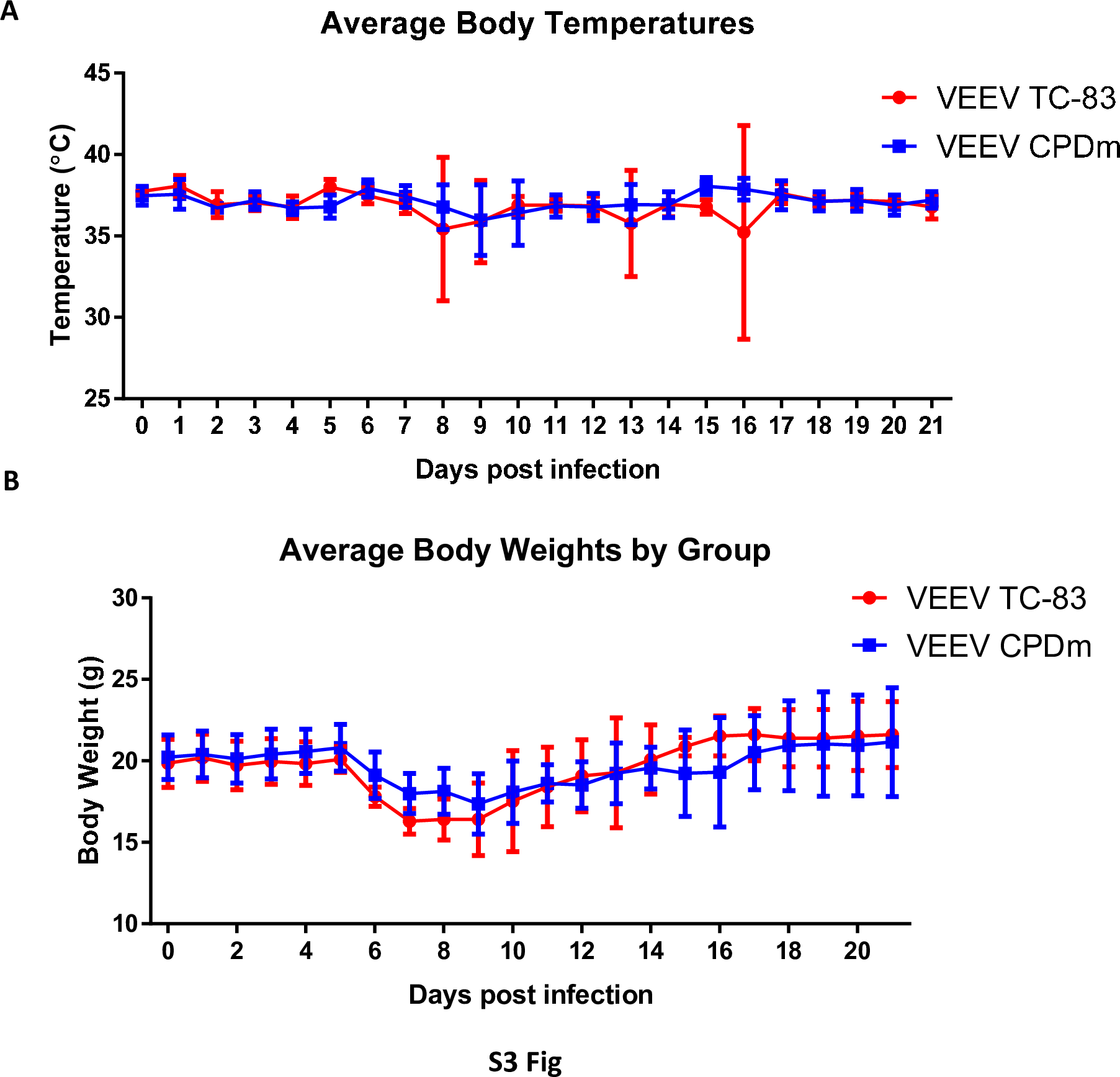

Supplement: S4 Fig — A) Daily average body temperature readings from mice infected with VEEV TC-83 or VEEV CPD. B) Daily average body weights from mice infected with VEEV TC-83 or VEEV CPD. (TIF) [file ppat.1008282.s004.tif]
